# Higher glucose fluctuation is associated with a higher risk of cardiovascular disease: Insights from pooled results among patients with diabetes

**DOI:** 10.1111/1753-0407.13386

**Published:** 2023-04-18

**Authors:** Feng Li, Lei Zhang, Yun Shen, Huan‐Huan Liu, Zhen‐Ye Zhang, Gang Hu, Ru‐Xing Wang

**Affiliations:** ^1^ Department of Cardiology Wuxi People's Hospital Affiliated to Nanjing Medical University Wuxi China; ^2^ Department of Endocrinology and Metabolism Shanghai Jiao Tong University Affiliated Sixth People's Hospital Shanghai China; ^3^ Pennington Biomedical Research Center Baton Rouge Louisiana USA

**Keywords:** cardiovascular disease, diabetes, glucose fluctuation, HbA1c variability, meta‐analysis, 心血管疾病, 糖尿病, 葡萄糖波动, HbA_1c_变异性, *meta*分析

## Abstract

**Background:**

The relationship between glucose fluctuation and the risk of cardiovascular disease (CVD) in patients with diabetes remains elusive. Glycated hemoglobin (HbA1c) variability is a key parameter of glucose fluctuation.

**Methods:**

PubMed, Cochrane Library, Web of Science, and Embase were searched up to 1 July 2022. Studies reporting associations of HbA1c variability (HbA1c‐SD), coefficient of variation of HbA1c (HbA1c‐CV), and HbA1c variability score [HVS] with the risk of CVD among patients with diabetes were included. We used three different insights (a high‐low value meta‐analysis, a study‐specific meta‐analysis, and a non‐linear dose–response meta‐analysis) to explore the relationship between HbA1c variability and CVD risk. A subgroup analysis was also performed to screen the potential confounding factors.

**Results:**

A total of 14 studies with 254 017 patients with diabetes were eligible. The highest HbA1c variability was significantly associated with increased risks of CVD (HbA1c‐SD, risk ratio [RR] 1.45; HbA1c‐CV, RR 1.74; HVS, RR 2.46; all *p* < .001) compared to the lowest HbA1c variability. The RRs of CVD for per HbA1c variability were significantly >1 (all *p* < .001). The subgroup analysis for per HbA1c‐SD found a significant exposure‐covariate interaction in the types of diabetes (*p* = .003 for interaction). The dose–response analysis showed a positive association between HbA1c‐CV and CVD risk (*P* for nonlinearity <.001).

**Conclusions:**

Our study suggests that the higher glucose fluctuation is significantly associated with the higher CVD risk in diabetes patients based on HbA1c variability. The CVD risk associated with per HbA1c‐SD might be higher among patients type 1 diabetes than patients with type 2 diabetes.

## Graphical abstract

**Highlights**

- This study is the first registered meta‐analysis with three different insights to explore the relationship between the glycated hemoglobin (HbA1c) variability and the risk of cardiovascular disease (CVD).
- Our study suggests that the higher glucose fluctuation is significantly associated with the higher CVD risk in diabetes patients based on HbA1c variability.
- The CVD risk associated with per HbA1c‐SD might be higher among patients type 1 diabetes than patients with type 2 diabetes.

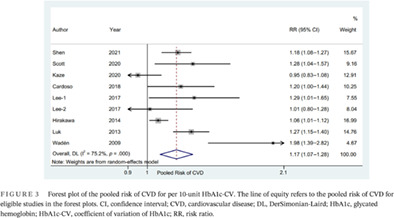

## 摘要

**背景:**血糖波动与糖尿病患者心血管疾病(CVD)风险的关系尚不清楚。糖化血红蛋白(HbA_1c_)变异性是反映血糖波动的关键指标。

**方法:**检索PubMed、Cochrane Library、Web of Science、Embase等数据库, 检索时间截至2022年7月1日。纳入HbA_1c_变异性指标[HbA_1c_标准差(HbA_1c_‐SD)、HbA_1c_变异系数(HbA_1c_‐ CV)和HbA_1c_变异性评分(HVS)]与糖尿病患者CVD风险关联的研究。我们使用了三种不同的方式(高低值*meta*分析、针对特定研究的*meta*分析和非线性剂量‐反应*meta*分析), 探讨HbA_1c_变异性指标与CVD风险之间的关系, 并进行亚组分析, 以筛选潜在的混杂因素。

**结果:**共有14项涉及254017名糖尿病患者的研究符合条件。相比于HbA_1c_变异性低的患者, HbA_1c_变异性越高与CVD风险增加显著相关(HbA_1c_‐SD, 风险比[RR] 1.45;HbA_1c_‐CV, RR 1.74;HVS, RR 2.46;所有P <0.001)。每HbA1c变异性导致CVD的RRs均显著大于1(均P <0.001)。每HbA_1c_‐SD亚组分析发现, 糖尿病类型的暴露‐协变量存在显著的相互作用(相互作用P = 0.003)。剂量‐反应分析显示HbA_1c_‐CV与CVD风险呈正相关(非线性P<0.001)。

**结论:**基于HbA_1c_变异性, 较高的血糖波动与糖尿病患者较高的CVD风险显著相关。与每HbA_1c_‐SD相关的CVD风险在1型糖尿病患者中可能高于2型糖尿病患者。

## 1 INTRODUCTION

Diabetes is one of the most common metabolic diseases in clinical practice, mainly including type 1 diabetes and type 2 diabetes. It has been reported that an estimated prevalence of diabetes would be over 300 million worldwide by 2025.^1^ The progression and deterioration of diabetes could lead to the development of multiple complications, including acute complications and long‐term complications, in which cardiovascular disease (CVD) has been widely concerned due to a significant association between CVD and diabetes‐related disability and mortality.^2^, ^3^

Glucose fluctuation, also named glucose variability, is defined as the variability of glucose homeostasis during a certain interval of time, including short‐term glucose fluctuation and long‐term glucose fluctuation.^4^, ^5^ Glycated hemoglobin (HbA1c) variability is a key parameter of glucose fluctuation. Accumulated studies revealed that several physiological and pathological factors, mainly including anemia, chronic kidney failure, pregnancy, age, ethnicity, and medications, might affect the measurement of HbA1c levels due to the changes of erythrocyte lifespan, alteration of hemoglobin glycosylation rate, and interference HbA1c detection.^6^

Multiple HbA1c variability indices based on the measurement of HbA1c were used to evaluate the glucose fluctuation, such as the SD of HbA1c (HbA1c‐SD), coefficient of variation of HbA1c (HbA1c‐CV), and HbA1c variability score (HVS).^7^ Accumulated evidence revealed that HbA1c variability might be associated with the development and progression of CVD. Moreover, animal studies indicated that multiple pathophysiological mechanisms (such as oxidative stress, inflammasome activation, endoplasmic reticulum stress, and cardiomyocyte apoptosis) might be involved in the glucose fluctuation induced CVD.^8^, ^9^, ^10^ However, challenges on clarification of the relationship between the HbA1c variability and CVD risk still remain unsolved owing to the limitation of study region, relatively small sample size, and difference of glucose fluctuation index. The aim of the present study was to assess the association between the HbA1c variability and the risk of CVD based on the pooled results among patients with diabetes.

## 2 METHODS

### 2.1 Study design

This study was performed according to the preferred reporting items for reviews and the Preferred Reporting Items for Systematic Reviews and Meta‐Analyses (PRISMA) guidelines. Our study protocol has been registered on the International Prospective Register of Systematic Reviews (PROSPERO) platform (registration number: CRD42022345008). The population, intervention, comparator, and outcomes (PICO) question is as follow: In the patients with diabetes, is higher glucose fluctuation (measured by HbA1c variability) associated with a higher risk of cardiovascular disease over a long‐term follow‐up (at least 12 months, mean or median) from clinical studies?

### 2.2 Study search strategy

A total of four online databases, including PubMed, Cochrane Library, Web of Science, and Embase, were comprehensively searched up to July 1, 2022 by two independent reviewers (FL and LZ). The searching keywords mainly included “type 2 diabetes,” “type 2 diabetes mellitus,” “T2DM,” “type 1 diabetes,” “type 1 diabetes mellitus,” “T1DM,” “HbA1c variability,” “visit‐to‐visit HbA1c variability,” “standard deviation of HbA1c,” “coefficient of variation of HbA1c,” “HbA1c SD,” “HbA1c CV,” “cardiovascular disease,” “CVD,” “cardiovascular events,” “cardiovascular endpoints,” “major adverse cardiovascular events,” and “MACE.” Trials investigating the association of HbA1c variability with the risk of CVD among patients with type 2 diabetes were included. The reference list of review literatures was also searched to screen the possible publications not being identified previously. Importantly, the corresponding authors were contacted for the missing key data in their publications (eg, the range of each HbA1c variability category and the corresponding cases number of cardiovascular disease).

### 2.3 Study selection criteria

The article titles, abstracts, and full texts were searched by two independent reviewers (FL and HHL) to identify the eligible studies. The disagreements about the eligibility would be resolved by a third reviewer (RXW). The inclusion criteria are as follows: (a) randomized controlled trials, prospective/retrospective cohorts, and observational studies; (b) studies investigating the association of HbA1c variability with the risk of CVD among patients with diabetes; (c) studies with full‐text published in peer‐reviewed journals; and (d) studies containing the latest data for multiple publications of the same study. Animal studies, review articles (including meta‐analysis), case reports, studies without original data, letters, and editorials were excluded.

### 2.4 Data extraction

Data from each eligible study were extracted by two independent researchers (LZ and HHL), and a third reviewer (RXW) was consulted to resolve any disagreements. We first extracted the study design and patient demographic characteristics, including first author, publication year, study region, numbers of patients, age, gender, comorbidity history (eg, CVD), duration of diabetes, and follow‐up time. Meanwhile, the clinical related indices, including HbA1c variability index, HbA1c measurements, the range of HbA1c variability in each HbA1c variability category, cases number and diabetic patients' number in each HbA1c variability category, effect size index, the definition of CVD, numbers of CVD, and adjusted variables were also documented.

### 2.5 Quality assessment

The study quality was assessed by two independent reviewers (FL and LZ) using the Newcastle‐Ottawa Quality Assessment Scale (NOS). The NOS contained three parts (including selection, comparability, and outcome) with nine points. When the score was >6, the quality of studies was considered as moderate‐to‐high quality; otherwise, the quality of studies was considered as low quality. Similarly, a third reviewer (RXW) was responsible for resolving the disagreements in the quality assessment process.

### 2.6 Statistical analysis

In our meta‐analysis, two effect size indices, including hazard ratio (HR) and odds ratio (OR), were displayed in the eligible studies. According to the previously reported methods,^11^ we used the risk ratio (RR) as the uniform effect size for pooled results because HR and OR were equivalent to RR. We used the Stata version 16.0 (College Station, TX USA, StataCorp LLC) for all statistical analyses. *p* < .05 was considered statistically significant.

We used a total of three different perspectives to assess the relationship between the HbA1c variability and the risk of CVD. First, a high‐low value meta‐analysis with the lowest and the highest HbA1c variability was performed. Second, similar with a reported method by Jayedi et al,^12^ a study‐specific meta‐analysis with estimated RR of CVD based on per 1‐unit HbA1c variability (including per 10‐unit HbA1c‐CV and per HbA1c‐SD) in each eligible study was performed. For this, RR and 95% confidence interval (CI) based on the per 1‐unit HbA1c variability were extracted. Otherwise, median points, the cases number of CVD, individual number (or person years), and corresponding RR and 95% CI in each category of HbA1c variability in each study were extracted for calculating estimated RR of CVD for per 1‐unit HbA1c variability based on a linear dose–response meta‐analysis. If a median point of HbA1c variability in each category was unavailable, the midpoint between the lower boundaries and upper boundaries was defined as the dose of each category. Moreover, if the highest HbA1c variability was open ended, a dose of 1.5 times the lower boundary was set as the midpoint of the highest HbA1c variability. Third, a nonlinear dose–response meta‐analysis using restricted cubic spline fit with four knots at fixed percentiles of the distribution (eg, 5%, 30%, 65%, and 95%) was performed to precisely, flexibly, and efficiently assess the association of the HbA1c variability with the risk of CVD. The *p* value for nonlinearity was evaluated via testing the null hypothesis that the coefficients of the second and the third spline were zero.

In addition, the Cochran Q test and I‐squared (I^2^) were used to estimate and quantify the heterogeneity among eligible studies. If the I^2^ value >50% or/and *p* < .05 for the Q test, we considered the heterogeneity significant, and a random effect model would be performed. Otherwise, a fixed effect model was used. A sensitivity analysis with sequentially omitting one study method was performed to evaluate the influence of a single study on the overall risk. The Egger's test was performed to assess the potential publication bias.

A subgroup analysis was also performed according to our previous reported methods.^13^ A total of seven confounding factors were screened, including study region (Asian and non‐Asian), numbers of patients with diabetes (≥10 000 and < 10 000), age (≥60 years and < 60 years), gender (≥50% male and <50% male), types of diabetes (type 2 diabetes and type 1 diabetes), adjustment of diabetes duration (Yes and No), adjustment of baseline HbA1c (Yes and No), and follow‐up time (≥5 years and <5 years).

## 3 RESULTS

### 3.1 Study selection and study characteristics

**FIGURE 1:**
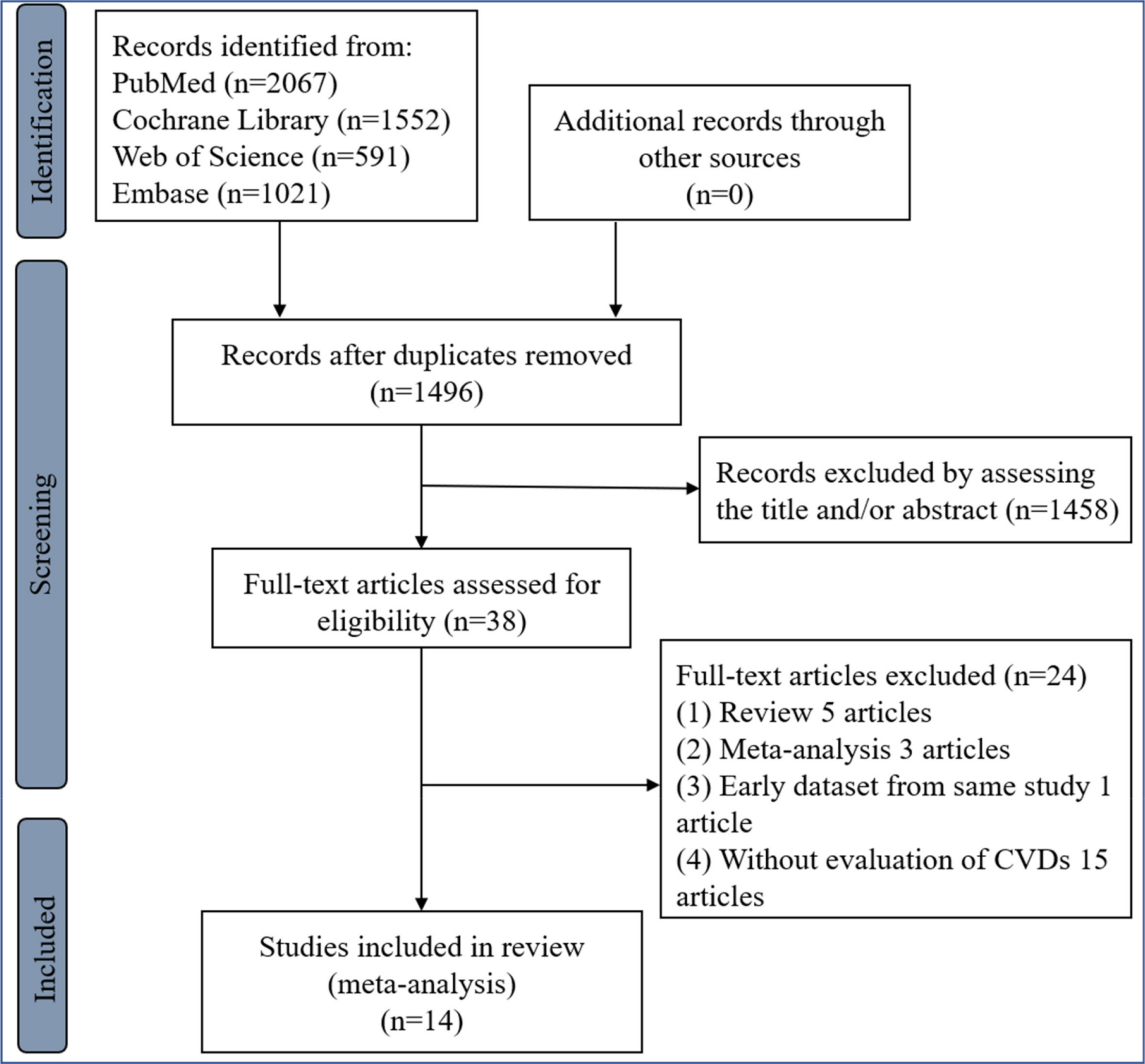
The flow chart of the study selection. CVD, cardiovascular disease.

A total of 14 studies^4^, ^5^, ^7^, ^14^, ^15^, ^16^, ^17^, ^18^, ^19^, ^20^, ^21^, ^22^, ^23^, ^24^ with 254 017 patients with diabetes (including 252 172 patients with type 2 diabetes from 13 studies^4^, ^5^, ^7^, ^15^, ^16^, ^17^, ^18^, ^19^, ^20^, ^21^, ^22^, ^23^, ^24^ and 1845 patients with type 1 diabetes from one study^14^) were eligible (Figure 1, Supplementary Data, Supplemantary text 1, and Supplemantary text 2). All eligible studies were the nonrandomized studies. According to the estimated glomerular filtration rate (eGFR) of 60 min/mL/1.73m^2^, one study was divided into two items for further analysis.^18^ Among our eligible studies, only one study used HVS to assess the HbA1c variability, which had been reported to be an emerging alternative for better clinical practice.^7^ Importantly, the range of each HbA1c variability category, the number of CVD cases, individual number (or person years), and corresponding effect size and 95% CI in each category of HbA1c variability could be available in three eligible studies.^4^, ^20^, ^24^ Per 1‐unit HbA1c variability (eg, per 10‐unit HbA1c‐CV) in two studies was calculated using a dose–response meta‐analysis with estimated RR of CVD.^20^, ^24^ The eligible study characteristics and the clinical related indices were displayed in Tables 1 and 2, respectively. Moreover, all our eligible studies showed a moderate‐to‐high quality (Supplementary Table S1).

**TABLE 1:** The baseline characteristics of the eligible studies.

First author	Year	Study design	Country	Total number of diabetes patients	Age (years)	Gender (male, %)	CVD history	Effect size index	Duration of diabetes (Years)	Follow‐up (Years)
Ceriello^5^	2022	Prospective cohort	Sweden	101 533	64.2 (median)	55.6	No	HR	>5 years (29.1%)	Median 4.4
Sato^24^	2021	Post‐hoc	Japan	4532	63.0 ± 10.6	47.5	No	OR	13.0 ± 8.8	Median 3.2
Shen^4^	2021	Retrospective cohort	United States	29 260	66.0 ± 11.6	45.9	No	HR	NA	Mean4.18
Scott^23^	2020	Post‐hoc	Australia	9790	62.3 ± 6.9	62.7	No	HR	4.3 ± 2.8	Mean 2.0
Li^7^	2020	Retrospective cohort	Scotland	21 352	63.3 ± 11.1	54.6	No	HR	NA	Median 6.8
Kaze^22^	2020	Post‐hoc	United States	3560	58.4 ± 6.7	37.9	No	HR	5.0 (2.0–9.0)	Median 6.8
Sun^21^	2019	Prospective cohort	China	930	64.8 ± 5.9	49.8	Had a major macrovascular/microvascular history	HR	7.8 ± 6.1	Median 4.8
Critchley^20^	2019	Retrospective cohort	United Kingdom	58 832	67.7 ± 10.9	55.4	No	HR	>5 years (37.5%)	Mean 4.1
Cardoso^19^	2018	Prospective cohort	Brazil	654	60.1 ± 9.6	38.1	Had a major macrovascular/microvascular history	HR	8.0 (3.0–15.0)	Median 9.3
Lee‐1^18^	2017	Retrospective cohort	Taiwan	6425	60.3 ± 11.4	51.5	No	HR	>5 years (91.7%)	Mean 6.3
Lee‐2^18^	2017	Retrospective cohort	Taiwan	1834	68.6 ± 10.9	54	No	HR	>5 years (92.6%)	Mean 6.3
Takao^17^	2015	Retrospective cohort	Japan	632	55.7 ± 9.3	82.1	No	HR	5.7 ± 6.7	Median 15.4
Hirakawa^16^	2014	Post‐hoc	Australia	4399	65.5 ± 6.3	42.6	Had a major macrovascular/microvascular history	HR	>5 years (56%)	Median 3.0
Luk^15^	2013	Prospective cohort	China	8439	57.6 ± 13.2	47	No (82.7%)	HR	6.0 (1.0–11.0)	Median 7.2
Wadén^14^	2009	Prospective cohort	Finland	1845	36.3 ± 11.8	53.3	No (91.4%)	HR	22.0 ± 11.8	Median 5.7

Abbreviations: CVD, cardiovascular disease; HR, hazard ratio; NA, not available; OR, odds ratio.

**TABLE 2:** The clinical related indices of the eligible studies.

First author	HbA1c measurements	CVD definition	Adjusted variables
Ceriello^5^	At least five measures during exposure phase (3 years)	Primary composite outcome: A composite of first occurrence of nonfatal myocardial infarction, nonfatal stroke, and all‐cause mortality.	Baseline variables: age, gender, duration of diabetes, body weight, smoking, values of HbA1c, systolic and diastolic blood pressure, total cholesterol, HDL, LDL, triglycerides, albuminuria, eGFR, retinopathy, treatment for diabetes, hypertension, dyslipidemia, and aspirin
Sato^24^	At least three HbA1c measurements	Composite cardiovascular endpoints: cardiac, cerebral, renal, and vascular events.	Mode 3: age, sex, body mass index, smoking habits, duration of diabetes, group of statin therapy, hypertension, diabetic nephropathy, diabetic neuropathy, estimated glomerular filtration rate, the number of HbA1c measurements + mean‐HbA1c.
Shen^4^	At least four HbA1c measurements obtained within 2 years of their first diagnosis of type 2 diabetes	Cardiovascular events including coronary heart disease and stroke	Mode 2: age, race, sex, smoking, body mass index, systolic blood pressure, non‐HDL/HDL ratio, eGFR, insurance type, hypoglycemia events, glucose‐lowering medications, anti‐hypertensive medications, lipid‐lowering medications, and antiplatelet and anticoagulant medications by category differences + the updated mean value of HbA1c
Scott^23^	History and physical examinations occurred at baseline and 4–6 monthly, with fasting venous blood (HbA1c, glucose) at baseline and annually, with treatment for diabetes management made by the regular treating clinician	Major cardiovascular disease events (coronary heart disease events, total stroke and other cardiovascular death)	Model 3 is adjusted for age, gender, baseline HbA1c or glucose, 2‐year HbA1c or glucose, fenofibrate or placebo allocation, systolic blood pressure, diabetes duration, prior cardiovascular disease, prior microvascular complications, baseline use of oral hypoglycemics, insulin, and antihypertensives.
Li^7^	At least five HbA1c measurements	Major adverse cardiovascular events	Full adjusted mode: sex, index age, calendar year, Scottish Index of Multiple Deprivation quintiles, ever smoking, hypertension at baseline, BMI at baseline, HDL cholesterol at baseline, eGFR at baseline, antiplatelet therapy at baseline, and Charlson Comorbidity Index+ time‐weighted average HbA1c
Kaze^22^	Variability of HbA1C was assessed using four indices across measurements from four study visits	CVD composite (myocardial infarction, stroke, hospitalization for angina, and CVD‐related deaths)	Model 3: age, sex, race/ethnicity, and randomization arm, body mass index, current smoking, alcohol drinking, use of antihypertensive medications, average ratio of total to high‐density lipoprotein cholesterol, estimated glomerular filtration rate, duration of diabetes, and average systolic blood pressure + average HbA1C.
Sun^21^	Visit‐to‐visit variability of HbA1c and FPG was evaluated using the five measurements at 3, 6, 12, 18, and 24 months, and measured as the standard deviation of HbA1c and FPG measurements.	MACE (major adverse cardiovascular events) was identified as occurrence of any of an episode of major macrovascular events and major microvascular events	Adjusted for age, gender, BMI, diabetes duration, baseline high‐ and low‐density lipoprotein cholesterol, triglyceride, albumin‐to‐creatinine ratio, baseline use of oral glucose‐lowering agents, baseline use of insulin, mean hemoglobin A1c or fast plasma glucose during the first 24 months, history of major macrovascular diseases, history of major microvascular diseases, and smoking status
Critchley^20^	At least one HbA1c measurement in each calendar year during the 4‐year baseline period	Cardiovascular‐related mortality	Full adjusted model: age, age*age, sex, duration of diabetes, index of multiple deprivation, smoking, and BMI, the number of HbA1c measures
Cardoso^19^	At least three annual HbA1c	Total cardiovascular events (CVEs: fatal or non‐fatal myocardial infarctions, sudden cardiac deaths, new‐onset heart failure, death from progressive heart failure, any myocardial revascularization procedure, fatal or nonfatal strokes, any aortic or lower limb revascularization procedure, any amputation above the ankle, and deaths from aortic or peripheral arterial disease)	Model 3: age, sex and number of HbA1c or FG measurements, diabetes duration, BMI, smoking status, physical inactivity, arterial hypertension, number of anti‐hypertensive drugs in use, ambulatory 24 h SBP, presence of micro‐ and macrovascular complications at baseline, serum mean HDL‐ and LDL‐cholesterol, and use of insulin, statins and aspirin, mean fasting glycemia and HbA1c
Lee‐1^18^	At least three HbA1c measurements	Cardiovascular events were defined as hospitalization for coronary artery disease, unstable angina, myocardial infarction, stroke, peripheral artery disease, and cardiovascular death	Age, sex, and significant variables, including mean HbA1C (per 1%), in the univariate analysis were used in the multivariate analysis
Lee‐2^18^	At least three HbA1c measurements	Cardiovascular events were defined as hospitalization for coronary artery disease, unstable angina, myocardial infarction, stroke, peripheral artery disease, and cardiovascular death	Age, sex, and significant variables, including mean HbA1C (per 1%), in the univariate analysis were used in the multivariate analysis
Takao^17^	At least four clinic visits	CVD events were defined as fatal or non‐fatal acute myocardial infarction, coronary artery procedure (bypass surgery or angioplasty), or stroke (ischemic or hemorrhagic), that required hospitalization	Clinical variables (mean HbA1c, mean SBP, number of visits (ln‐transformed), age, sex, diabetes duration, BMI, TC/HDL cholesterol, eGFR, baseline smoking status, baseline alcohol intake, baseline use of insulin, and baseline use of antihypertensive agents), HbA1c‐CV, and SBPCV
Hirakawa^16^	Five measurements of HbA1c	Major macrovascular events were defined as death from cardiovascular causes, nonfatal myocardial infarction, or nonfatal stroke.	Age, sex, randomized blood pressure lowering, region, duration of diabetes, baseline smoking status, baseline alcohol intake, systolic blood pressure, total cholesterol, log‐transformed triglycerides, BMI, baseline use of oral glucose‐lowering agents, baseline use of insulin, and mean HbA1c, or FBG during the first 24 months
Luk^15^	The frequency of performing HbA1c was at the discretion of the attending physicians with considerable variations.at least one repeat measurement of HbA1c during the observation period	CVD was defined as events of myocardial infarction (ICD‐9 code 410), ischemic heart disease (ICD‐9 code 411–414), heart failure (ICD‐9 code 428), nonfatal ischemic stroke (ICD‐9 code 432–434 and 436) or peripheral vascular disease (ICD‐9 code 440.2, 440.4 and443.9)	Adjusted for age, gender, smoking history, diabetes duration, body mass index, waist circumference, systolic/diastolic blood pressures, LDL cholesterol, HDL cholesterol, log triglyceride, log urine ACR, estimated GFR, hemoglobin and baseline medication use including the use of ACE inhibitors/ARB, antihypertensive drugs, lipid‐lowering drugs, oral hypoglycemic drugs, and insulin.
Wadén^14^	The median number of A1C measurements per patient was 13 (interquartile range 7–20), that is 2.3 measurements per patient and year	CVD was defined based on medical records both at baseline and at follow‐up as any of the following hard events: myocardial infarction, coronary artery procedure (bypass surgery or angioplasty), stroke (ischemic or hemorrhagic), limb amputation because of ischemia, or a peripheral artery procedure	Duration of diabetes, sex, systolic blood pressure, total cholesterol, ever smoking, intrapersonal mean of serial A1C measurements, and number (ln‐transformed) of A1C measurements. For incident CVD events, we additionally adjusted for the presence of diabetic nephropathy and CVD events at baseline.

Abbreviations: ACE, angiotensin‐converting enzyme; ARB, angiotensin receptor blocker; BMI, body mass index; CVD, cardiovascular disease; eGFR, estimated glomerular filtration rate; FBG, fasting blood glucose; HbA1c, glycated hemoglobin; HbA1c‐CV, coefficient of variation of HbA1c; HDL, high‐density lipoprotein; ICD‐9, International Classification of Diseases, Ninth Revision; LDL, low‐density lipoprotein; NA, not available; SBP, systolic blood pressure; TC, total cholesterol.

### 3.2 A high‐low value meta‐analysis

**FIGURE 2:**
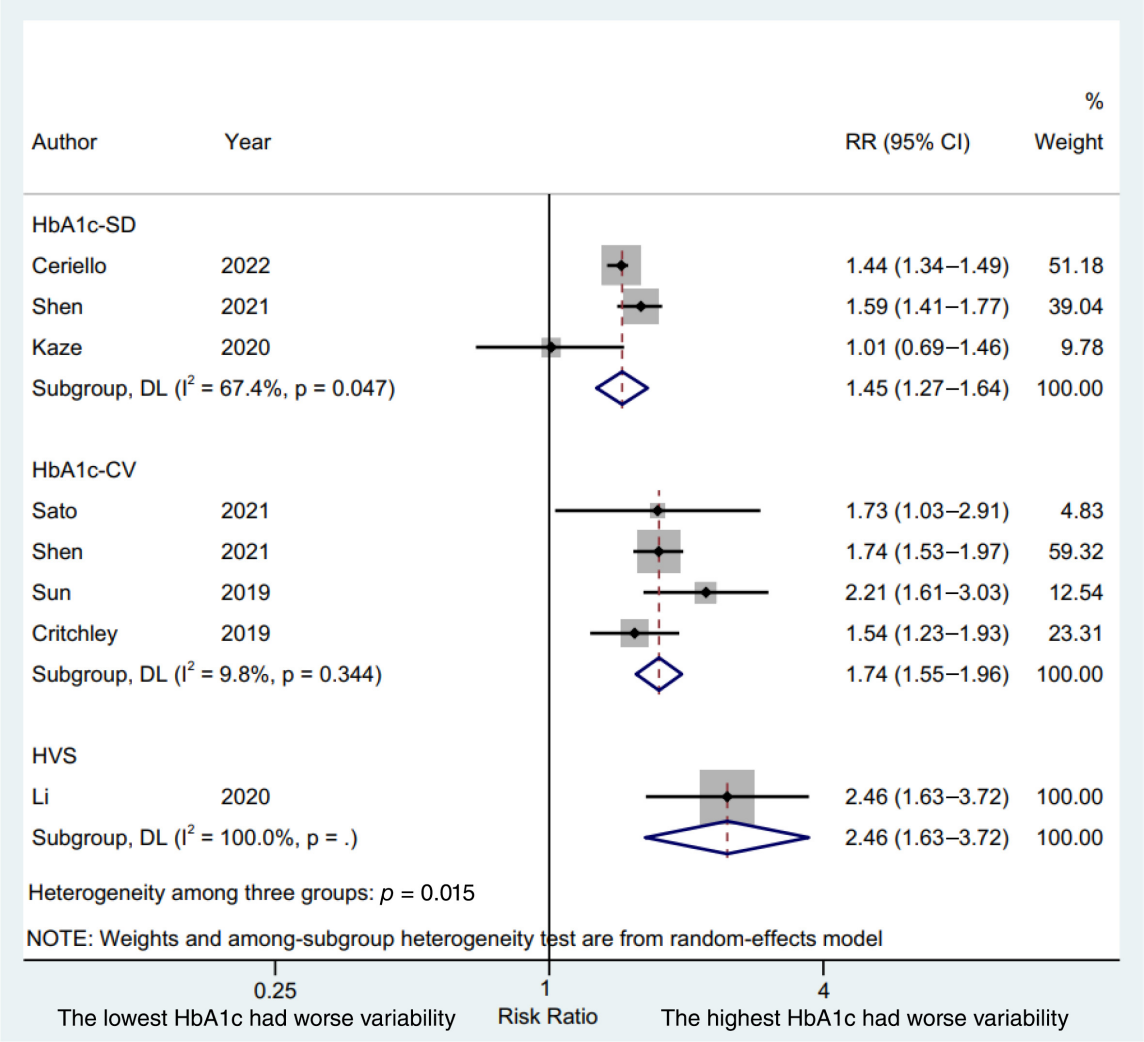
Forest plot of the risk of cardiovascular disease between the lowest and the highest HbA1c variability. CI, confidence interval; DL, DerSimonian‐Laird; HbA1c, glycated hemoglobin; HbA1c‐CV, coefficient of variation of HbA1c; HVS, HbA1c variability score; RR, risk ratio.

We used the lowest and the highest HbA1c variability to conduct a high‐low value meta‐analysis. A total of three studies,^4^, ^5^, ^22^ four studies,^4^, ^7^, ^21^, ^24^ and one study^20^ reported the lowest and the highest HbA1c variability with the HbA1c‐SD, HbA1c‐CV, and HVS, respectively, as the HbA1c variability index. The results were presented in Figure 2. Patient with the highest HbA1c variability indices were significantly associated with an increased risks of CVD compared with patients with the lowest HbA1c variability indices (HbA1c‐SD, RR 1.45, 95% CI 1.27–1.64, *p* = .000, I^2^ = 67.4%; HbA1c‐CV, RR 1.74, 95% CI 1.55–1.96, *p* = .000, I^2^ = 9.8%; HVS, RR 2.46, 95% CI 1.63–3.72, *p* = .000, I^2^ = 100.0%; *P* for interaction = .015) with a random effect model. The RR of CVD was higher in terms of HbA1c‐CV and HVS compared to HbA1c‐SD (*p* = .035 and *p* = .016, respectively), whereas a similar RR of CVD was displayed between HbA1c‐CV and HVS (*p* = .114).

### 3.3 A study‐specific meta‐analysis

**FIGURE 3:**
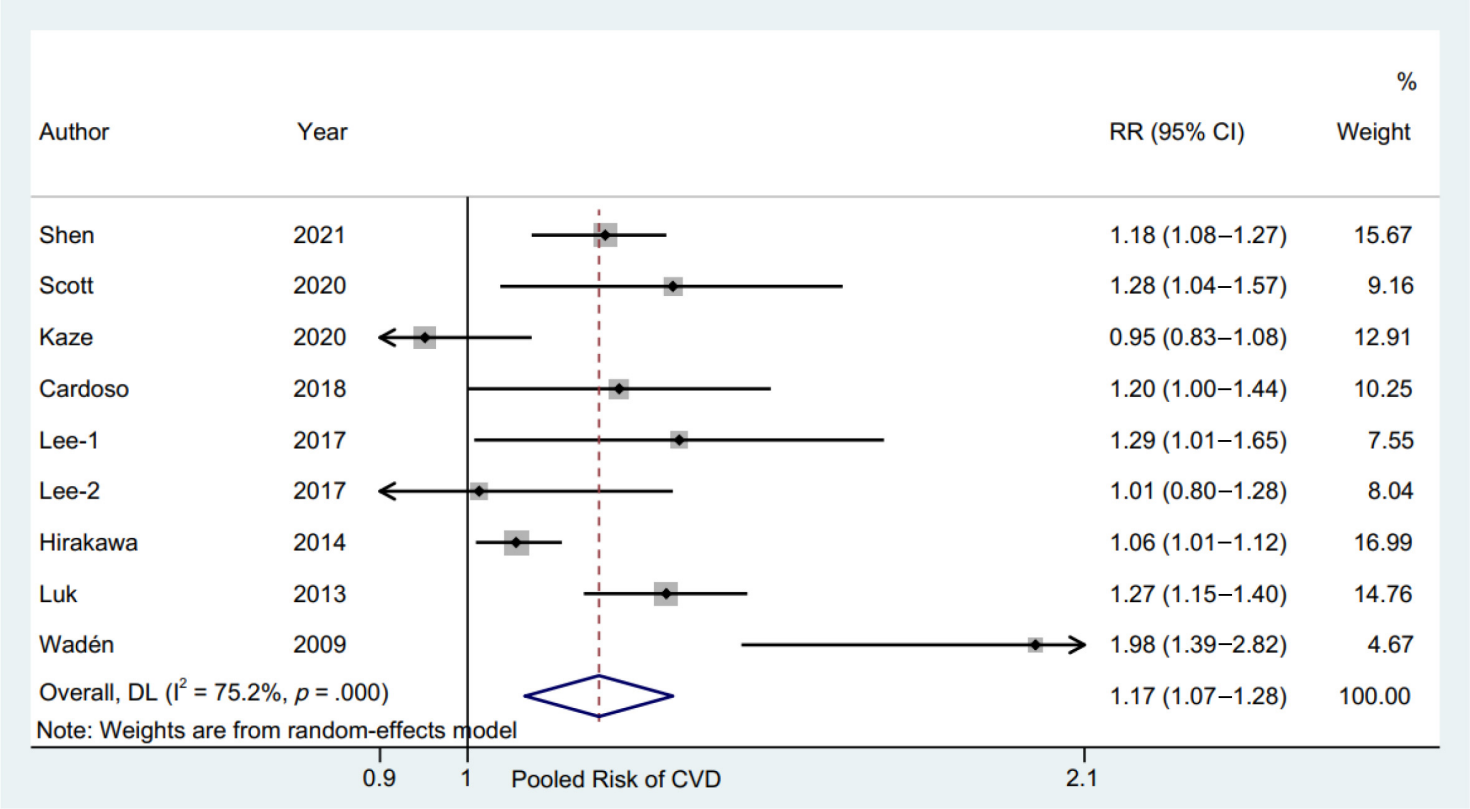
Forest plot of the pooled risk of CVD for per 10‐unit HbA1c‐CV. The line of equity refers to the pooled risk of CVD for eligible studies in the forest plots. CI, confidence interval; CVD, cardiovascular disease; DL, DerSimonian‐Laird; HbA1c, glycated hemoglobin; HbA1c‐CV, coefficient of variation of HbA1c; RR, risk ratio.

**FIGURE 4:**
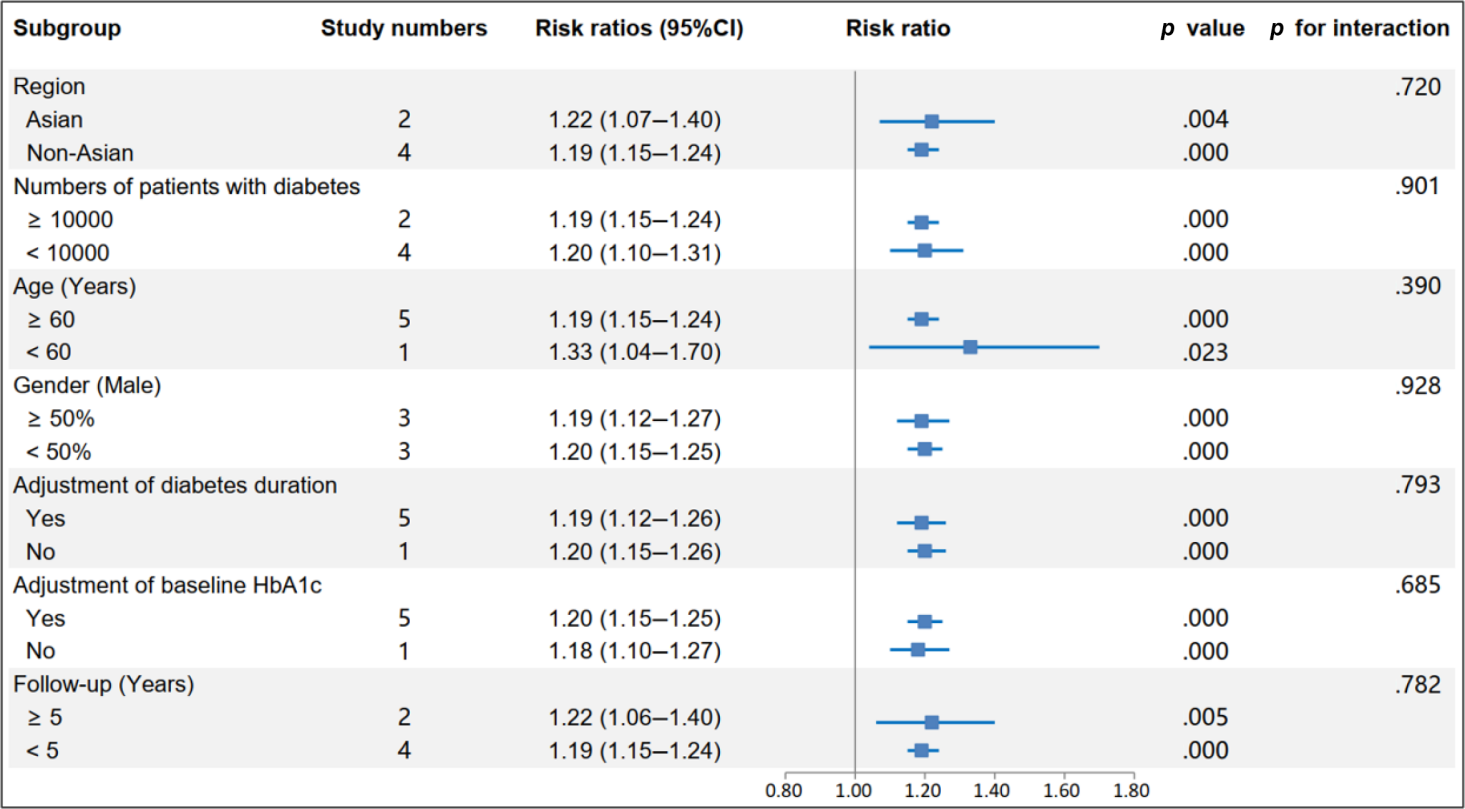
Subgroup analysis of the risk of cardiovascular disease for per 10‐unit HbA1c‐CV. Subgroup analysis was performed based on seven confounding factors. CI, confidence interval; HbA1c, glycated hemoglobin; HbA1c‐CV, coefficient of variation of HbA1c.

The RR and corresponding 95% CI of per 10‐unit HbA1c‐CV were available from six eligible studies,^4^, ^17^, ^19^, ^20^, ^23^, ^24^ and per HbA1c‐SD was also available from seven studies^4^, ^15^, ^16^, ^18^, ^19^, ^22^, ^23^ of patients with type 2 diabetes and one study^14^ of patients with type 1 diabetes. The pooled RR of CVD for per 10‐unit HbA1c‐CV was 1.20 (95% CI 1.15–1.24, *p* = .000, I^2^ = 0.0%) with a random effect model (Figure 3). The sensitivity analysis indicated no significant change in the overall combined proportion, ranging from 1.19 (95% CI, 1.12–1.26) to 1.20 (95% CI, 1.15–1.25). Meanwhile, Egger's test showed no potential publication bias (*p* = .662). These results suggested that our pooled results were robust. In addition, the subgroup analysis for seven confounding factors showed a positive association between per 10‐unit HbA1c‐CV and CVD risk (Figure 4).

**FIGURE 5:**
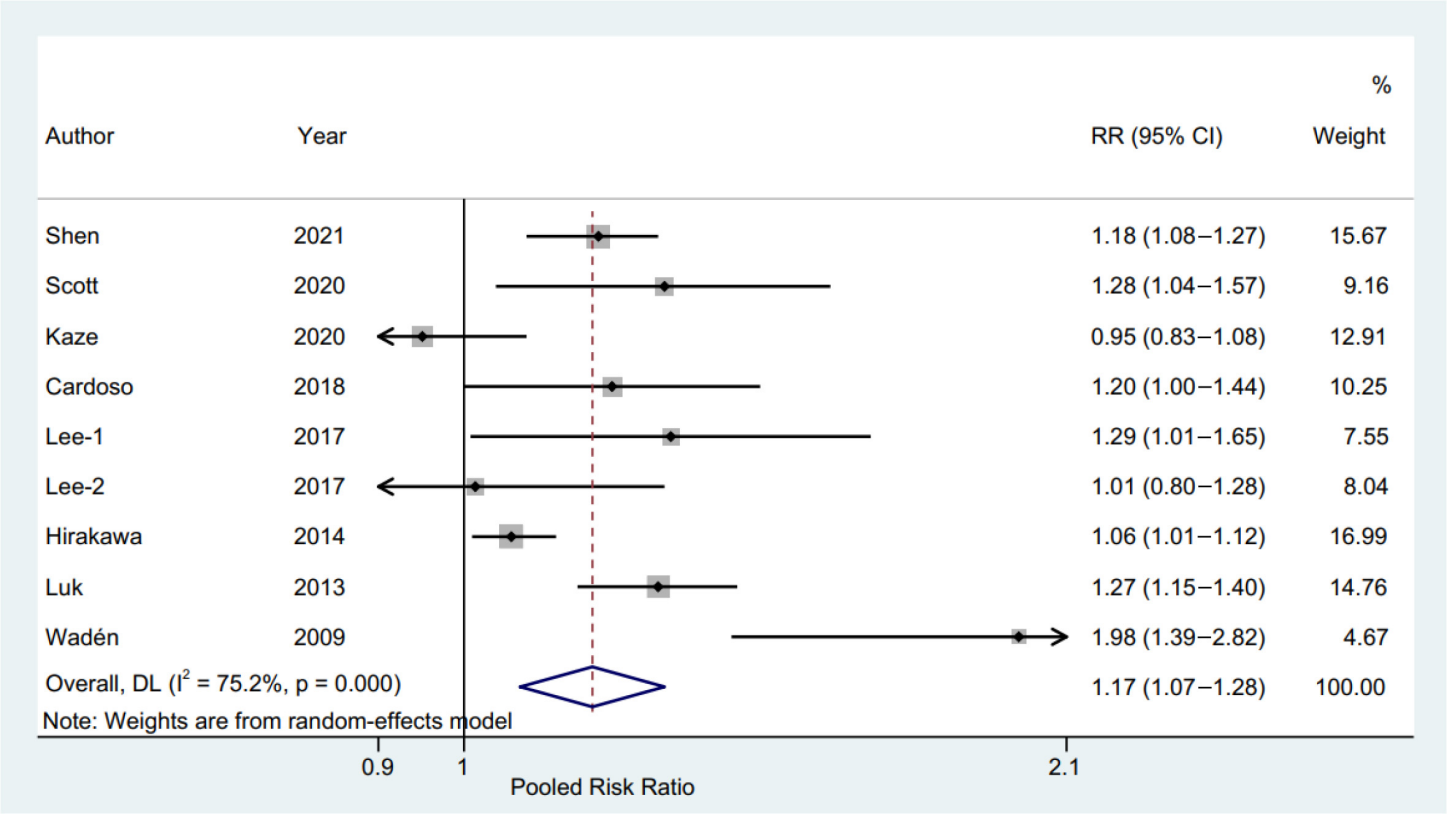
Forest plot of the pooled risk of cardiovascular diseases for per HbA1c‐SD. The line of equity refers to the pooled risk ratio of eligible studies in the forest plots. CI, confidence interval; DL, DerSimonian‐Laird; HbA1c, glycated hemoglobin; RR, risk ratio.

The pooled results with random effect model showed that RR of CVD for per HbA1c‐SD was 1.12 (95% CI 1.08–1.16, *p* = .000, I^2^ = 75.2%) with a random effect model (Figure 5). The sensitivity analysis found that RRs ranged from 1.14 (95% CI, 1.05–1.23) to 1.21 (95% CI, 1.10–1.32), and the Egger's test also showed no potential publication bias (*p* = .179).

**FIGURE 6:**
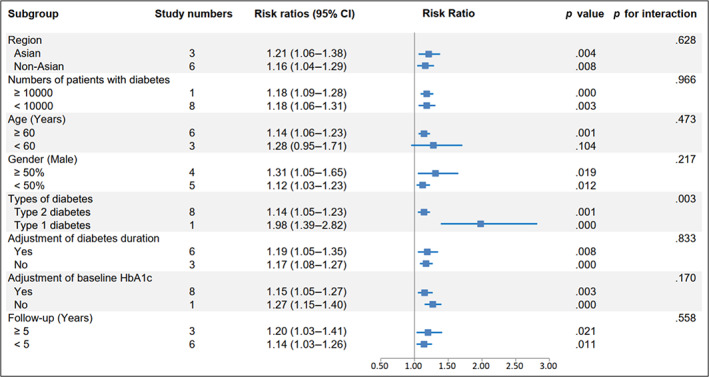
Subgroup analysis of the risk of cardiovascular disease for per HbA1c‐SD. Subgroup analysis was performed based on seven confounding factors. CI, confidence interval; HbA1c, glycated hemoglobin.

Importantly, a significant exposure‐covariate interaction was identified in the types of diabetes subgroup, including type 2 diabetes subgroup (RR 1.14; 95% CI, 1.05–1.23, *p* = .001) and type 1 diabetes subgroup (RR 1.98; 95% CI, 1.39–2.82, *p* = .000) with *p* = 0.003 for interaction. Moreover, in the age subgroup, the RR was 1.14 (95% CI, 1.06–1.23, *p* = .001) in the ≥60 years arm, while the RR was 1.28 (95% CI, 0.95–1.71, *p* = .104) in the other arm. The remaining subgroup results were consistent with the pooled result, indicating a higher risk CVD trend (Figure 6).

### 3.4 A non‐linear dose–response meta‐analysis

**FIGURE 7:**
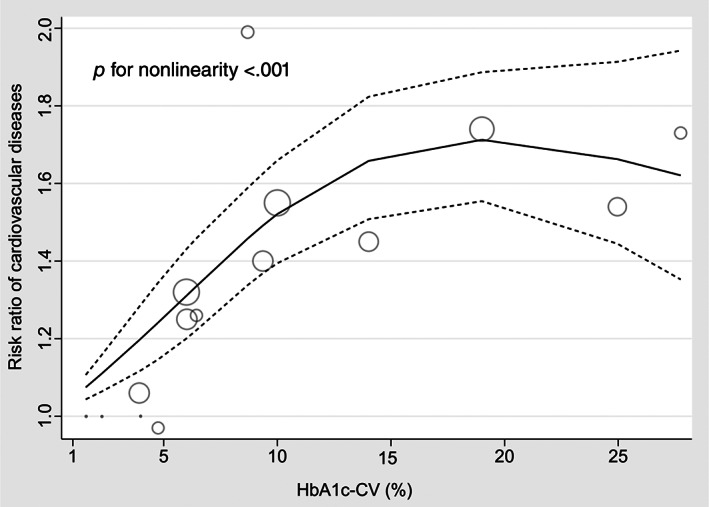
Restricted cubic spline fit between HbA1c‐CV and the cardiovascular disease risk. A dose–response association between HbA1c‐CV and the cardiovascular disease risk. The solid black line represents nonlinear dose response. The dotted black line represents the 95% confidence interval. Gray circles represent risk ratio point estimates for HbA1c‐CV with circle size proportional to inverse of standard error. The small gray points represent the referential category of the HbA1c‐CV in each study. HbA1c, glycated hemoglobin; HbA1c‐CV, coefficient of variation of HbA1c.

Only three studies^4^, ^20^, ^24^ were available for the dose–response analysis between HbA1c‐CV and the risk of CVD among patients with type 2 diabetes. The results of the dose–response meta‐analysis were displayed in Figure 7, which indicated that there was a positive association between HbA1c‐CV at range from 1.57% to 14.02% and the risk of CVD, whereas a relatively high RR level of CVD risk was maintained in the HbA1c‐CV range from 14.02% to 27.75% (*P* for nonlinearity <.001). The RR of CVD according to the dose of HbA1c‐CV among patients with type 2 diabetes was displayed in Supplementary Table S2.

## 4 DISCUSSION

The present meta‐analysis indicated that large HbA1c variability was associated with an increased risk of CVD among patients with diabetes, and this positive association was higher among patients with type 1 diabetes than those with type 2 diabetes.

Glucose fluctuation, also termed glucose variability, is defined as the variability of glucose homeostasis during a certain interval of time, including short‐term glucose fluctuation (eg, within−/between‐days glucose variability) and long‐term glucose fluctuation (eg, between‐weeks/months glucose variability).^4^, ^5^ At present, multiple indices were reported to represent the variability of glucose homeostasis in clinic, including HbA1c‐SD, HbA1c‐CV, and HVS. HbA1c‐SD was the SD of HbA1c value with several measurements of HbA1c, meanwhile the ratio of HbA1c‐SD and HbA1c mean value represented the HbA1c‐CV. Moreover, HVS, an emerging index for glucose fluctuation, is the HbA1c variability score, reflecting the frequent decreases or increases by >0.5%. Li et al^7^ reported that, compared with the lowest quintile, HVS > 60% was associated with the elevated risk of macrovascular and microvascular complications in the newly diagnosed type 2 diabetes patients, which was expected to be a more superior and translatable alternative for evaluating the glucose fluctuation. Interestingly, all three indices were available in our eligible studies.

Accumulated clinical studies revealed that glucose fluctuation was significantly associated with the increasing risk of cardiovascular‐related disease. A Swedish national diabetes register study including 101 533 patients with type 2 diabetes showed that higher HbA1c variability was associated with an increased risk of CVD, which suggested that optimal management for patients with type 2 diabetes might be achieved by the effective control of HbA1c variability.^5^ A recent meta‐analysis with 12 eligible studies also indicated that long‐term glucose fluctuation might serve as a significantly independent risk factor for cardiovascular events among patients with type 2 diabetes.^25^ In addition, an increasingly growing number of animal studies explored the potential mechanisms between glucose fluctuation and cardiovascular‐related diseases and events. Multiple studies showed that oxidative stress played a key role in the development and progression of atherosclerosis due to the glucose fluctuation.^26^, ^27^ Moreover, our team found that glucose fluctuation could increase reactive oxygen species overproduction and activate the protein kinase C alpha/nuclear factor kappa B subunit 1/muscle ring‐finger protein‐1 (PKCα/NF‐κB/MuRF1) signaling to promote vascular large conductance calcium activated potassium channel dysfunction, ultimately leading to coronary artery diastolic dysfunction.^8^ Recently, our team also found glucose fluctuation not only aggravated myocardial fibrosis via activation of the nucleotide‐binding oligomerization domain‐like receptor protein 3^9^ but also promoted cardiomyocyte apoptosis due to trigger endoplasmic reticulum stress.^10^

Challenges still existed for drawing a consistent conclusion in clinic due to the limitation of study region, relatively small sample size, and difference of glucose fluctuation index. Therefore, we comprehensively enrolled a total of 254 017 patients with type 2 diabetes from 14 original studies. To the best of our knowledge, this study is the first registered meta‐analysis to find a consistent result of all three HbA1c variability indices (HbA1c‐SD, HbA1c‐CV, and HVS) with increased risks of CVD among patients with type 2 diabetes. Interestingly, consistent with previous studies,^7^, ^28^ the risk of CVD was higher in terms of HbA1c‐CV and HVS compared to HbA1c‐SD, suggesting that HbA1c‐CV and HVS might be better indices for evaluating the CVD risk among patients with type 2 diabetes. Similarly, a study‐specific meta‐analysis also showed the RR of CVD was >1 in terms of both per 10‐unit HbA1c‐CV and per HbA1c‐SD. Subgroup analysis results for most of the subgroup confounding factors were consistent with the pooled results.

Type 1 diabetes and type 2 diabetes, the two major types of diabetes in clinic, have been effectively controlled due to the deep understanding of pathological mechanisms of diabetes and the advances in hypoglycemic drugs. However, a recent study based on the Australian diabetes registry database reported that the incidence of hypoglycemia remained stable among patients with type 1 diabetes but was significantly decreased among patients with type 2 diabetes during the 10‐year follow‐up.^29^ This result suggested that hypoglycemia might not be effectively controlled for patients with type 1 diabetes, which facilitated worse glycemia control, ultimately leading to an increased risk of CVD.^30^ Meanwhile, a higher glucose fluctuation was more likely to occur among type 1 diabetes because of the absolute insulin deficiency and inappropriate use of hypoglycemic drugs.^31^, ^32^ Moreover, Shen et al^4^ also highlighted that hypoglycemia played a significant mediating role between HbA1c variability and the incidence of CVD. Similarly, our subgroup analysis for per HbA1c‐SD showed that the risk of CVD was significantly higher among patients with type 1 diabetes than those with type 2 diabetes, which might be attributed to a vulnerably worse glycemia control, a high incidence of hypoglycemia, and the functional loss of islet cells among type 1 diabetes.

Additionally, the elderly patients with diabetes were vulnerable to suffering from the dysfunction of adaptive physiologic responses, multiple chronic conditions, and hypoglycemia, leading to a poor prognosis.^33^ Interestingly, we found that in terms of per HbA1c‐SD, the significantly increased risk of CVD was shown in the ≥60 years subgroup, whereas that was not shown in the <60 years subgroup, which partly indicated a higher risk of CVD in the elder patients with diabetes. However, the same trends were presented in both age subgroups in terms of per 10‐unit HbA1c‐CV. Therefore, more studies were needed to further demonstrate our age subgroup results.

Importantly, a dose–response analysis showed that there was a positive association between HbA1c‐CV and the CVD risk, and a relatively high risk of CVD was maintained with the increase of HbA1c‐CV. This result was consistent with the high‐low value meta‐analysis and the study‐specific meta‐analysis in our study, indicating our results were relatively reliable.

### 4.1 Limitations

Several limitations in this study should be highlighted. First, a total of 14 studies with patients with diabetes were eligible and analyzed in our study, indicating our results were applicable only to patients with diabetes and not to individuals with impaired fasting glucose, impaired glucose tolerance, and normal blood glucose. Second, the measurement of HbA1c levels might be affected by multiple physiological and pathological factors (including changes of erythrocyte lifespan, alteration of hemoglobin glycosylation rate, and interference with HbA1c detection), leading to some potential biases influencing our results.^6^ However, the HbA1c variability was determined via multiple HbA1c measurements taken over a period of years in each eligible studies, which could significantly improve the reliability and accuracy of the HbA1c variability. Moreover, three different insights (including a high‐low value meta‐analysis, a study‐specific meta‐analysis, and a nonlinear dose–response meta‐analysis) were performed, showing the consistent results on the relationship between the HbA1c variability and the risk of CVD. Meanwhile, the sensitivity analysis and Egger's test both indicated that our results were robust. Third, three studies with the HbA1c‐CV for the dose–response analysis revealed that a positive association between HbA1c‐CV and the CVD risk is shown and a relatively high risk of CVD was maintained with the increase of HbA1c‐CV. However, a relatively small sample size might affect our nonlinear fit between HbA1c‐CV and the risk of CVD ultimately letting us fail to draw a substantial conclusion. Moreover, the results of the dose–response analysis may not be extended to patients with type 1 diabetes because the patients in the three eligible studies all suffer from type 2 diabetes. Fourth, a minority of patients with diabetes in several eligible studies had a history of CVD. Whereas, all studies performed multivariate models to avoid to potentially overestimate the pooled CVD risk. Finally, only one study with patients with type 1 diabetes was eligible in the subgroup analysis of different types of diabetes. Similarly, relative fewer eligible studies made the types of diabetes subgroup result to be interpreted with more caution. Therefore, more prospective and longitudinal follow‐up studies were needed to validate our results.

### 4.2 Conclusion

Our study suggests that the higher glucose fluctuation is significantly associated with the higher CVD risk among in diabetes patients based on HbA1c variability. The CVD risk associated with HbA1c‐SD might be higher among patients type 1 diabetes than patients with type 2 diabetes.

## AUTHOR CONTRIBUTIONS

Ru‐Xing Wang developed the concept of the study; Feng Li, Lei Zhang, and Yun Shen designed this study and carried out the data analysis; Feng Li wrote the manuscript with help from Lei Zhang, Yun Shen, Huan‐Huan Liu, and Zhen‐Ye Zhang; Ru‐Xing Wang and Gang Hu provided critical reviews of the paper. All authors have read and approved the final manuscript.

## FUNDING INFORMATION

This work was supported by the Natural Science Foundation of China (81770331). Dr. Hu was partly supported by the grant from the National Institute of General Medical Sciences (U54GM104940).

## CONFLICT OF INTEREST STATEMENT

The authors declare that there is no conflict of interests regarding the publication of this paper.

## Supporting information


**Data S1.** Supporting Information.

**Data S2.** Supporting Information.

**Data S3.** Supporting Information.

**Table S1.** Quality assessment of eligible studies according to the Newcastle‐Ottawa Quality Assessment Scale.
**Table S2.** Risk ratios (95% CI) of cardiovascular disease in patients with type 2 diabetes from nonlinear dose–response analysis according to HbA1c‐CV.

## Data Availability

The data that support the findings of this study are available from the corresponding author upon reasonable request.
