# Supplementary material for: Higher glucose fluctuation is associated with a higher risk of cardiovascular disease: Insights from pooled results among patients with diabetes
Source: J Diabetes. 2023 Apr 18;15(5):368–81. doi: 10.1111/1753-0407.13386 (PMC10172020; doi:10.1111/1753-0407.13386)
Supplement: Supplementary file 2 — Data S2. Supporting Information. [file JDB-15-368-s004.docx]

**The Search Strategy in PubMed**

#1 "Diabetes Mellitus"[MeSH Terms] OR "Diabetes Mellitus, Type 1"[All Fields] OR "Diabetes Mellitus, Type 2" [All Fields] OR "T2DM"[All Fields] OR "T1DM"[All Fields] OR "Type 2 Diabetes"[All Fields] OR "Type 1 Diabetes"[All Fields]

#2 "Glucose Fluctuation "[All Fields] OR "HbA1c variability"[All Fields] OR "standard deviation of HbA1c"[All Fields] OR "coefficient of variation of HbA1c"[All Fields] OR "HbA1c SD" [All Fields] OR " HbA1c CV" [All Fields]

#3 "Cardiovascular Diseases"[MeSH Terms] OR " Cardiovascular Disease"[All Fields] OR "Disease, Cardiovascular"[All Fields] OR " Major Adverse Cardiac Events"[All Fields] OR "MACE" [All Fields] OR " Cardiac Events" [All Fields] OR " Cardiac Event" [All Fields] OR " Event, Cardiac" [All Fields] OR " Adverse Cardiac Event" [All Fields] OR " Adverse Cardiac Events" [All Fields] OR " Cardiac Event, Adverse" [All Fields] OR " Cardiac Events, Adverse" [All Fields] OR " Cardiovascular Events" [All Fields] OR " Cardiovascular Endpoints" [All Fields]

#4 #1 AND #2 AND #3
