# Supplementary material for: Higher glucose fluctuation is associated with a higher risk of cardiovascular disease: Insights from pooled results among patients with diabetes
Source: J Diabetes. 2023 Apr 18;15(5):368–81. doi: 10.1111/1753-0407.13386 (PMC10172020; doi:10.1111/1753-0407.13386)
Supplement: Supplementary file 3 — Data S3. Supporting Information. [file JDB-15-368-s002.docx]

**Full-text articles excluded (n=24)**

**1. Review 5 articles**

10.1002/dmrr.3047

10.1136/bmjdrc-2020-002032

10.1016/j.numecd.2012.03.006

10.1007/s11886-012-0332-4

10.1159/000446471

**2. Meta-analysis 3 articles**

10.1055/a-1730-4904

10.2337/dc15-1188

10.1055/a-1730-5029

**3. Early dataset from same study 1article**

10.1016/j.jdiacomp.2014.02.006

**4. Without evaluation of CVDs 15 articles**

10.1016/j.jdiacomp.2012.03.028

10.1136/bmjdrc-2014-000060

10.1007/s00125-012-2816-6

10.2337/dc08-0864

10.1371/journal.pone.0091137

10.2337/dc10-2028

10.5603/EP.2014.0012

10.1016/j.amjmed.2013.04.015

10.3122/jabfm.2011.04.100186

10.1111/1753-0407.12403

10.1007/s00125-012-2700-4

10.2337/dc12-2264

10.1186/1475-2840-12-98

10.1111/j.1464-5491.2012.03767.x

10.1007/s00125-012-2572-7
