# Supplementary material for: Higher glucose fluctuation is associated with a higher risk of cardiovascular disease: Insights from pooled results among patients with diabetes
Source: J Diabetes. 2023 Apr 18;15(5):368–81. doi: 10.1111/1753-0407.13386 (PMC10172020; doi:10.1111/1753-0407.13386)
Supplement: Supplementary file 4 — Table S1. Quality assessment of eligible studies according to the Newcastle‐Ottawa Quality Assessment Scale. Table S2. Risk ratios (95% CI) of cardiovascular disease in patients with type 2 diabetes from nonlinear dose–response analysis according to HbA1c‐CV. [file JDB-15-368-s003.docx]

**Supplementary Table 1.** Quality assessment of eligible studies according to the Newcastle-Ottawa Quality Assessment Scale

| First author | Year | Selection |  |  |  | Comparability | Outcome |  |  | Total stars |
| --- | --- | --- | --- | --- | --- | --- | --- | --- | --- | --- |
|  |  | Representativeness of the exposed cohort | Selection of the nonexposed cohort | Ascertainment of exposure | Demonstration that outcome of interest was not present at start of study | Comparability of cohorts on the basis  of the design or analysis | Assessment of outcome | Was follow-up long enough for outcomes to occur | Adequacy of follow-up of cohorts |  |
| Ceriello^[5]^ | 2022 | **☆** | **☆** | **☆** | **☆** | **☆☆** | **☆** | **☆** | **☆** | 9 |
| Sato^[24]^ | 2021 |  | **☆** | **☆** | **☆** | **☆** | **☆** | **☆** | **☆** | 7 |
| Shen^[4]^ | 2021 | **☆** | **☆** | **☆** | **☆** | **☆** |  | **☆** | **☆** | 7 |
| Scott^[23]^ | 2020 |  | **☆** | **☆** | **☆** | **☆☆** |  | **☆** | **☆** | 7 |
| Li^[7]^ | 2020 | **☆** | **☆** | **☆** | **☆** | **☆** | **☆** | **☆** | **☆** | 8 |
| Kaze^[22]^ | 2020 |  | **☆** | **☆** | **☆** | **☆☆** | **☆** | **☆** | **☆** | 8 |
| Sun^[21]^ | 2019 | **☆** | **☆** | **☆** | **☆** | **☆** | **☆** |  | **☆** | 7 |
| Critchley^[20]^ | 2019 | **☆** | **☆** | **☆** | **☆** | **☆** | **☆** |  | **☆** | 7 |
| Cardoso^[19]^ | 2018 | **☆** | **☆** | **☆** | **☆** | **☆** | **☆** | **☆** | **☆** | 8 |
| Lee-1^[18]^ | 2017 | **☆** | **☆** | **☆** | **☆** | **☆☆** | **☆** | **☆** | **☆** | 9 |
| Lee-2^[18]^ | 2017 | **☆** | **☆** | **☆** | **☆** | **☆☆** | **☆** | **☆** | **☆** | 9 |
| Takao^[17]^ | 2015 | **☆** | **☆** | **☆** | **☆** | **☆☆** | **☆** | **☆** | **☆** | 9 |
| Hirakawa^[16]^ | 2014 |  | **☆** | **☆** | **☆** | **☆** | **☆** |  | **☆** | 6 |
| Luk^[15]^ | 2013 | **☆** | **☆** | **☆** | **☆** | **☆☆** | **☆** | **☆** | **☆** | 9 |
| Wadén^[14]^ | 2009 | **☆** | **☆** | **☆** | **☆** | **☆☆** | **☆** | **☆** | **☆** | 9 |

**Supplementary Table 2.** Risk ratios (95% CI) of cardiovascular disease in patients with type 2 diabetes from non-linear dose–response analysis according to HbA1c-CV

| HbA1c-CV (%) | Cardiovascular disease risk |
| --- | --- |
| 1.57 | 1.07 (1.04-1.11) |
| 2.27 | 1.11 (1.06-1.16) |
| 3.93 | 1.20 (1.12-1.29) |
| 4.00 | 1.20 (1.12-1.29) |
| 4.76 | 1.24 (1.15-1.34) |
| 6.00 | 1.31 (1.20-1.43) |
| 6.02 | 1.31 (1.20-1.43) |
| 6.44 | 1.34 (1.22-1.46) |
| 8.70 | 1.46 (1.34-1.59) |
| 9.37 | 1.49 (1.37-1.63) |
| 10.00 | 1.52 (1.39-1.66) |
| 14.02 | 1.66 (1.51-1.82) |
| 19.00 | 1.71 (1.55-1.89) |
| 24.96 | 1.66 (1.44-1.91) |
| 27.75 | 1.62 (1.35-1.94) |

HbA1c-CV: coefficient of variation of HbA1c; CI: confidence interval
